# Early use of tocilizumab in solid organ transplant recipients with COVID‐19: A retrospective cohort study in Saudi Arabia

**DOI:** 10.1002/iid3.587

**Published:** 2022-01-14

**Authors:** Amani H. Yamani, Basem M. Alraddadi, Reem S. Almaghrabi, Afnan A. Amer, Fatimah S. Mehdawi, Mohammed A AL‐Hamzi, Meshari S. Aldajani, Majda S. Alattas, Aiman M. Elsaed Ramadan, Ghassan Y Wali, Abeer N. Alshukairi, Abbas Al Mutair

**Affiliations:** ^1^ Department of Medicine, Division of Infectious Diseases King Faisal Specialist Hospital and Research Center Jeddah Saudi Arabia; ^2^ Department of Medicine, Division of Infectious Diseases King Faisal Specialist Hospital and Research Center Riyadh Saudi Arabia; ^3^ Infectious Diseases Department Fakeeh Care Jeddah Saudi Arabia; ^4^ Department of Medicine, Division of Infectious Diseases King Abdulaziz Specialist Hospital Taif Saudi Arabia; ^5^ Pharmaceutical Care Division King Faisal Specialist Hospital and Research Center Jeddah Saudi Arabia; ^6^ Infection Control Department King Abdulaziz Medical City Riyadh Saudi Arabia; ^7^ College of Medicine AlFaisal University Riyadh Saudi Arabia; ^8^ Research Center Almoosa Specialist Hospital Al‐ahsa Saudi Arabia; ^9^ College of Nursing Princess Nourah Bint Abdulrahman University Riyadh Saudi Arabia; ^10^ School of Nursing University of Wollongong Wollongong Australia

**Keywords:** COVID‐19, cytokine syndrome, SOT, tocilizumab

## Abstract

**Background:**

Tocilizumab was studied to reduce cytokine syndrome in patients with severe COVID‐19 pneumonia in solid organ transplant (SOT) recipients with conflicting results. We aim to study the early use of tocilizumab in SOT with COVID‐19 pneumonia on low flow oxygen.

**Methods:**

This is a retrospective cohort study that was conducted in two transplant centers in Saudi Arabia among 46 SOT with COVID‐19 comparing 21 patients who received tocilizumab to 25 patients who received standard of care. Their clinical characteristics and outcomes were described.

**Results:**

Compared to patients who received standard of care, patients in the tocilizumab group were older (60.2 ± 12.8 vs. 48.6 ± 12.3, *p* = .003), had higher ferritin (862.1 ± 919.1 vs. 414 ± 447.3, *p* = .025) and C‐reactive protein (CRP) (85 ± 83.1 vs. 42.9 ± 57.3, *p* = .012). More patients in the tocilizumab group required high flow oxygen (38.1% vs. 8.0%, *p* = .028) compared to patients on standard of care. There were no differences in mortality or mechanical ventilation requirement. Hospital stay was significantly shorter in the tocilizumab group than the standard of care group (9.6 ± 7.4 vs. 20.7 ± 11.7, *p* < .001).

**Conclusions:**

Early use of tocilizumab in SOT was associated with a shorter hospital stay. There was no difference in mortality rate and the requirement for mechanical ventilation in both groups.

## Graphical abstract

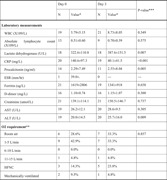

**Abbreviations**
ALT: alanine aminotransferase
ARDS: acute respiratory distress syndrome
AST: aspartate aminotransferase
ATG: anti‐thymocyte globulin
COVID‐19: corona virus disease 2019
CRP: C‐reactive protein
CRS: cytokine release syndrome
CXR: chest x‐ray
ESR: erythrocyte sedimentation rate
GGT: gamma glutamyltrsansferase
HFNC: high flow nasal cannula
ICU: intensive care unit
IL‐6: interleukin 6
IVIG: intravenous immune globulin
LDH: lactate dehydrogenase
LFT: liver function test
PCR: polymerase chain reaction
SARS‐CoV‐2: sever acute respiratory syndrome coronavirus 2
SD: standard deviation
SOT: solid organ transplant
WBC: white blood cell

## 1 INTRODUCTION

The current acute respiratory syndrome coronavirus 2 (SARS‐CoV‐2) caused the COVID‐19 pandemic.^1^, ^2^ Patients with mild to moderate symptoms constitute the majority of COVID‐19 cases and up to 20% may develop severe illness including pneumonia, acute respiratory distress syndrome, and multiorgan failure requiring ventilatory support.^3^ The same pattern of COVID‐19 severity was reported in Saudi Arabia with various clinical presentations including mild, moderate, and critical cases; in addition to asymptomatic cases.^4^, ^5^, ^6^, ^7^, ^8^

Previous studies showed that COVID‐19 disease is associated with an increase in plasma concentrations of cytokines and inflammatory markers including elevation of ferritin, lactate dehydrogenase (LDH), d‐dimer, and C‐reactive protein (CRP).^9^, ^10^, ^11^ Additionally, lung tissue histopathology examination of COVID‐19 deceased patients showed inflammatory cellular infiltration, proteinaceous exudate, and evidence of extensive alveolar edema.^3^, ^9^, ^10^ Such findings indicate that COVID‐19 is associated with pulmonary inflammation and cytokine storm secondary to dysregulated host immune response.^9^, ^10^

A systemic review reported overall mortality of 18.6% in solid organ transplant patients infected with COVID‐19.^11^ However, the mortality was 59% in patients admitted to the intensive care unit (ICU) in a cohort of 90 COVID‐19 solid organ transplant patients with variable disease severity.^12^ Severe COVID 19 pneumonia is associated with complex immune dysregulation, lymphopenia, macrophage activation syndrome, and elevated interleukin‐6 (IL‐6).^13^ Tocilizumab is a recombinant humanized anti‐IL‐6 receptor monoclonal antibody studied to reduce the cytokine syndrome in patients with severe COVID‐19 disease.^14^ Several retrospective case‐control studies and randomized trials evaluated the role of tocilizumab in reducing the mortality of severe COVID‐19 and had conflicting results.^7^, ^8^, ^9^, ^10^, ^11^, ^15^, ^16^, ^17^, ^18^, ^19^, ^20^ However, SOT recipients were not included in these studies except for one study, which only included nine cases (6%).^21^

The largest cohort of COVID 19 transplant patients who received IL‐6 blockers included 80 cases in Spain. The mortality rate was 32%. Patients who died continued to have an elevation of CRP despite receiving tocilizumab. The level of IL‐6 was not useful to assess response to tocilizumab, and it did not correlate with mortality.^22^ In a matched case–control study among COVID‐19 solid organ transplant recipients, there was no mortality difference between those receiving tocilizumab and those who received standard of care.^13^ Most patients had severe disease and required high flow oxygen and mechanical ventilation. Herein, we describe a cohort of COVID‐19 solid organ transplant recipients comparing patients who received tocilizumab to patients who received standard of care. We aimed to determine the impact of early use of tocilizumab in SOTs who are not ventilated and require low flow oxygen.

### 1.1 Definitions and eligibility

SARS‐Cov‐2 infection, defined as presenting with a fever or any respiratory symptoms, including dry cough, and especially in those with a history of travel or exposure to infected people within two weeks before the onset of illness since January 2020. Case definitions of confirmed human infection with SARS‐Cov‐2 were in accordance with the interim guidance from the WHO.^23^ Only patients with a laboratory‐confirmed infection by real‐time polymerase chain reaction (PCR) were enrolled in this study.

In the cases group of the current study, we included all patients with SARS‐CoV‐2 who received tocilizumab during the study period in addition to the standard of care. The control group of the current study included patients who were admitted to the hospital at the same study period but never received tocilizumab and were treated with the standard treatment only, as they were not eligible to receive tocilizumab.

### 1.2 Main outcome measures

Variables included patients' demographics, co‐morbid conditions, signs and symptoms of SARS‐CoV‐2 illness, chest radiological findings, laboratory abnormalities, microbiological testing, use of mechanical ventilation, ventilator modes and settings, interventions used to treat refractory hypoxemia, vasoactive support, medications offered to the patient and treatment outcomes (i.e., hospitalization, transferred, died, or discharged) on hospital admission, during patient's ICU stay, and at hospital discharge. Information sources were medical files, electronic health information records, and laboratories reports of COVID‐19 patients.

## 2 METHODS

### 2.1 Study design and patients

This is a retrospective cohort study conducted in two transplant centers in Saudi Arabia. The study population was adults (≥18 years) COVID‐19 solid organ transplant recipients who were admitted to King Faisal Specialist Hospital and Research Center, Jeddah (March 1, 2020, to October 31, 2020) and Riyadh (March 1, 2020, to July 31, 2020). COVID‐19 SOT recipients who received tocilizumab were compared to COVID‐19 hospitalized SOT patients who only received standard care. Patients were followed during hospitalization until death or discharge. Diagnosis of COVID‐19 was confirmed with a positive SARS‐CoV‐2 nasopharyngeal PCR. Both treatment groups who received tocilizumab and standard of care were diagnosed and managed at the same period time between May and September 2020.

We utilized the Research Electronic Data Capture (REDCap), a web‐based software tool that allows researchers to create secure online forms for data capture, management, and analysis; developed by Vanderbilt University (Nashville, TN, USA).^24^ We gathered the following parameters: patients' demographics, comorbid conditions, signs and symptoms of COVID‐19 illness, laboratory data, use of mechanical ventilation, ventilator settings and modes, medications, and treatment outcomes. This study was approved by the Institutional Review Board at King Faisal Specialist Hospital and Research Center in Jeddah, Saudi Arabia. They deemed it a low‐risk study and waived the need to obtain informed consent.

### 2.2 Treatment plan

According to the hospital guidelines that both institutions share, all COVID‐19 patients received standard of care treatment at hospital admission. Standard of care treatment in the current study included the use of both off‐label hydroxychloroquine and azathioprine or favipiravir and convalescent plasma. Later on, dexamethasone was added as part of standard care management. It also included oxygen supply to target oxygen saturation reaching at least 90%. patients received antibiotics at the discretion of the treating physician when suspecting a bacterial respiratory super‐infection. The majority of patients had their antimetabolite drugs held during admission.

Patients were eligible to receive tocilizumab if they had abnormal chest imaging consistent with COVID‐19 pneumonia, rapidly worsening respiratory symptoms/signs, or any requirement of supplemental O_2_ in the absence of systemic bacterial or fungal infection and increasing inflammatory parameters defined by any of the following: Ferritin > 500 μg/L or doubling within 24 h, CRP > 70 mg/L. The average dose of tocilizumab given was 4–8 mg/kg (recommend dose was 400 mg once) to be repeated after 12–24 h if the response to the first dose was inadequate (maximum of two doses with a single maximum dose of 800 mg). The decision of when to give tocilizumab was left to the discretion of the treating infectious diseases physician based on the above criteria.

### 2.3 Analytical approach

Baseline clinical characteristics on admission were compared between the two groups, including information related to their transplant, type of immunosuppression, history of any recently treated rejections in the year before COVID‐19 diagnosis, symptoms on presentation, oxygen requirements, chest X‐ray findings, and pertinent laboratory markers including complete blood count, renal profile, hepatic profile, cardiac profile and inflammatory markers including erythrocyte sedimentation rate (ESR), CRP, d‐Dimer, LDH, and ferritin and admission floor (isolation unit vs. ICU).

hospital course was compared between the two groups, including the requirement for high flow oxygen, mechanical ventilation, inotropic support, renal replacement therapy, and the development of secondary infections, modification of immunosuppressive therapy, use of COVID 19 targeted therapy, and death.

Inflammatory parameters (CRP, ferritin, and D dimer) and oxygen requirements were evaluated on Days 0 and 3 post‐tocilizumab infusion.

### 2.4 Data analysis

All categorical variables were presented as frequencies and percentages, while continuous variables were presented as means and standard deviations (SDs). Demographics and clinical characteristics on admission and disease course and outcomes were compared between the patients who received and did not receive tocilizumab. *χ* ^2^ or Fisher's exact test, as appropriate, were used to examine differences in categorical variables, while student *t* test or Mann–Whitney test, as appropriate, were used to examine differences in continuous variables. Wilcoxon signed ranks test was used to compare changes in laboratory markers and oxygen requirements between days 0 and 3 after receiving tocilizumab. All *p* values were two‐tailed. *p *< .05 was considered significant. SPSS software (release 25.0; IBM Corp) was used for all statistical analyses.

## 3 RESULTS

A total of 46 patients were included in the study; 21 patients in the tocilizumab group and 25 patients in the control group. As shown in Table 1, the average age was 53.9 ± 13.7 years, and 55.6% of the patients were males. The most frequent comorbidities were hypertension (63.0%), chronic kidney disease (47.8%), diabetes (41.3%), and coronary artery disease (15.6%). The most frequent transplanted organ was the kidney (78.3%), followed by liver (15.2%), heart (4.3%), and lung (2.2%). The majority (70.5%) of the patients received an organ from a living donor. Frequent maintenance immunosuppressive medications included prednisone (91.3%), tacrolimus (87.0%), and mycophenolate mofetil (82.6%). A total of four (8.7%) patients had a history of rejection in the year before COVID‐19 diagnosis, with methylprednisolone and rituximab the most frequently used medications. The most frequent COVID‐19 symptoms at the time of admission were fever (82.6%), cough (67.4%), diarrhea (47.8%), shortness of breath (46.7%), fatigue (41.3%), abdominal pain (28.3%), sore throat (27.8%), and myalgia (26.1%).

**Table 1:** Baseline demographic and clinical characteristics on admission

Age in years*	Tocilizumab group (*N* = 21)	Control group (*N* = 25)	Total (*N* = 46)	*p* value
Age in years*	60.2 ± 12.8	48.6 ± 12.3	53.9 ± 13.7	.003
Gender				
Male	14 (66.7%)	11 (45.8%)	25 (55.6%)	.161
Female	7 (33.3%)	13 (54.2%)	20 (44.4%)	
Body mass index (BMI)	29.85 ± 5.41	29.71 ± 5.82	29.77 ± 5.59	.93
Comorbidities				
Hypertension	15 (71.4%)	14 (56.0%)	29 (63.0%)	.280
Diabetes	10 (47.6%)	9 (36.0%)	19 (41.3%)	.425
Chronic kidney disease	12 (57.1%)	10 (40.0%)	22 (47.8%)	.246
Coronary artery disease	5 (23.8%)	2 (8.3%)	7 (15.6%)	.225
Chronic liver disease	3 (14.3%)	1 (4.0%)	4 (8.7%)	.318
Malignant neoplasm	1 (4.8%)	3 (12.0%)	4 (8.7%)	.614
Chronic pulmonary disease	1 (4.8%)	0 (0.0%)	1 (2.2%)	.457
Asthma	1 (4.8%)	0 (0.0%)	1 (2.2%)	.467
Transplanted organ				
Kidney	14 (66.7%)	22 (88.0%)	36 (78.3%)	.428
Liver	5 (23.8%)	2 (8.0%)	7 (15.2%)	
Heart	1 (4.8%)	1 (4.0%)	2 (4.3%)	
Lung	1 (4.8%)	0 (0.0%)	1 (2.2%)	
Type of donor				
Alive	11 (55.0%)	20 (83.3%)	31 (70.5%)	.040
Deceased	9 (45.0%)	4 (16.7%)	13 (29.5%)	
Symptoms				
Fever	17 (81.0%)	21 (84.0%)	38 (82.6%)	>.99
Cough	14 (66.7%)	17 (68.0%)	31 (67.4%)	.923
Runny nose	1 (5.0%)	5 (20.0%)	6 (13.3%)	.205
Sore throat	6 (31.6%)	4 (23.5%)	10 (27.8%)	.717
Shortness of breath	12 (57.1%)	9 (37.5%)	21 (46.7%)	.188
Chest pain	3 (14.3%)	1 (4.2%)	4 (8.9%)	.326
Myalgia	5 (23.8%)	7 (28.0%)	12 (26.1%)	.747
Fatigue	7 (33.3%)	12 (48.0%)	19 (41.3%)	.314
Headache	5 (23.8%)	4 (16.0%)	9 (19.6%)	.711
Loss of taste	0 (0.0%)	2 (8.0%)	2 (4.3%)	.493
Anosmia	0 (0.0%)	2 (8.0%)	2 (4.3%)	.493
Abdominal pain	7 (33.3%)	6 (24.0%)	13 (28.3%)	.484
Nausea	6 (28.6%)	5 (20.0%)	11 (23.9%)	.497
Vomiting	6 (28.6%)	5 (20.0%)	11 (23.9%)	.497
Diarrhea	8 (38.1%)	14 (56.0%)	22 (47.8%)	.226
Laboratory tests*				
WBC (×109/L)	5.60 ± 3.02	5.76 ± 2.94	5.69 ± 2.94	.741
Absolute lymphocyte count (×109/L)	0.76 ± 0.45	1.07 ± 0.73	0.92 ± 0.62	.162
Lactate dehydrogenase (U/L)	284.9 ± 117.6	206.6 ± 47.5	247.0 ± 97.8	.019
CRP (mg/L)	85.0 ± 83.1	42.9 ± 57.3	63.0 ± 73.0	.012
Procalcitonin (ng/ml)	0.30 ± 0.31	0.16 ± 0.17	0.23 ± 0.26	.151
Ferritin (μg/L)	862.1 ± 919.1	414.0 ± 447.3	627.9 ± 739.3	.025
d‐dimer (mg/L)	1.36 ± 1.33	0.78 ± 0.88	1.07 ± 1.15	.061
Creatinine (umol/L)	151.9 ± 134.0	138.1 ± 100.5	144.4 ± 115.8	.766
AST (U/L)	26.9 ± 13.1	21.3 ± 13.3	24.0 ± 13.3	.085
ALT (U/L)	23.6 ± 26.1	22.8 ± 20.0	23.1 ± 22.7	.563
ALP (U/L)	128.7 ± 201.0	87.5 ± 41.8	106.7 ± 140.4	.617
Bilirubin (umol/L)	8.3 ± 5.7	7.0 ± 3.0	7.6 ± 4.4	.980
Albumin (g/L)	35.7 ± 4.1	40.0 ± 3.7	38.1 ± 4.4	.001
GGT (IU/L)	297.4 ± 444.7	30.6 ± 2.1	186.3 ± 356.0	.048
Chest X‐ray				
Normal	4 (19.0%)	17 (68.0%)	21 (45.7%)	.003
Unilateral infiltrate	9 (42.9%)	3 (12.0%)	12 (26.1%)	
Bilateral infiltrate	8 (38.1%)	5 (20.0%)	13 (28.3%)	
O_2_ requirements				
Room air	18 (85.7%)	24 (96.0%)	42 (91.3%)	.595
1–5 L/min	1 (4.8%)	1 (4.0%)	2 (4.3%)	
6–10 L/min	1 (4.8%)	0 (0.0%)	1 (2.2%)	
HFNC	1 (4.8%)	0 (0.0%)	1 (2.2%)	
Admission location				
Isolation floor	16 (76.2%)	25 (100.0%)	41 (89.1%)	.015
Intermediate care	1 (4.8%)	0 (0.0%)	1 (2.2%)	
ICU*	4 (19.0%)	0 (0.0%)	4 (8.7%)	

*Note*: Data were presented as number and percentage unless mentioned otherwise.

Abbreviations: ALT, alanine aminotransferase; AST, aspartate aminotransferase; ATG, anti‐thymocyte globulin; COVID‐19, Corona Virus Disease 2019; CRP, C‐reactive protein; GGT, gamma glutamyltrsansferase; HFNC, High flow nasal cannula; ICU, intensive care unit; IVIG, intravenous immune globulin; WBC, white blood cell.

* Mean and standard deviation.

Laboratory examinations demonstrated the following mean and SD values 5.69 ± 2.94 white blood cell (WBC) count (×109/L), 0.92 ± 0.62 absolute lymphocyte count (×109/L), 205.0 ± 69.3 platelets count (×109/L), 247.0 ± 97.8 LDH (U/L), 63.0 ± 73.0 CRP (mg/L), 627.9 ± 739.3 ferritin (μg/L), 1.07 ± 1.15 d‐dimer (mg/L) (refer to Table 1).

More than half of the patients had lung infiltrates on chest X‐rays. Table 1 outlines clinical, laboratory, and radiological findings in patients in both groups. A total of 4 (8.7%) patients required oxygen; 1–5 L/min in two patients, 6–10 L/min in one patient, and high flow nasal cannula (HFNC) in one patient. The majority (89.1%) of the patients were admitted to the isolation floor, while 8.7% required admission to COVID‐19 designated ICU beds on presentation.

Treatment groups who received tocilizumab and standard of care were diagnosed and managed at the same period time between May and September 2020. Data analysis showed no statistical differences between the two groups when compared over time (*p* ≥ .05). In the tocilizumab group, the first dose of tocilizumab was administered on average after 6.5 ± 4.4 days from diagnosis with COVID‐19. Compared to the standard of care group, tocilizumab group had older patients (60.2 ± 12.8 vs. 48.6 ± 12.3, *p* = .003), more likely to receive transplant from a deceased donor (45.0% vs. 16.7%, *p* = .040), required more ICU/intermediate care admission (23.8% vs. 0.0%, *p* = .015), had higher levels of LDH (*p* = .019), CRP (*p* = .012), and ferritin (*p* = .025).

As shown in Table 2, frequent immunosuppression management during patient hospitalization included holding antimetabolite (79.1%), reducing calcineurin inhibitors (41.9%), and increasing baseline steroids (30.2%). Frequent COVID‐19 targeted therapy used included dexamethasone (48.6%), favipiravir (34.9%), azithromycin (32.6%), and hydroxychloroquine (23.3%). Of the patients included in the study in the two groups, a total of 12 (26.1%) had a bacterial infection (mainly pneumonia), and only one patient (2.2%) had candidemia. The average length of stay was 12.0 ± 9.2 days. Approximately 19.6% of patients required mechanical ventilation, 21.7% required HFNC, 6.5% required renal replacement therapy, 11.1% required inotropes/vasopressors, and 32.6% required ICU stay. A total of four (8.7%) patients had hospital death. Compared with the standard of care group, the tocilizumab group was more likely to use hydroxychloroquine (44.4% vs. 8.0%, *p* = .009), and azithromycin (50.0% vs. 20.0%, *p* = .038) as COVID‐19 targeted therapy. Compared with the standard of care group, the tocilizumab group was more likely to require HFNC (38.1% vs. 8.0%, *p* = .028) and ICU stay (52.4% vs. 16.0%, *p* = .009) and to have shorter hospitalization (9.6 ± 7.4 vs. 20.7 ± 11.7 days, *p* < .001). There were no differences between groups regarding hospital death or requirements of mechanical ventilation, renal replacement therapy, and inotropes/vasopressors use.

**Table 2:** Hospital course and outcome

	Tocilizumab group (*N* = 21)	Control group (*N* = 25)	Total (*N* = 46)	*p* value
Immunosuppression management during admission				
Antimetabolite held	14 (77.8%)	20 (80.0%)	34 (79.1%)	>.99
Calcineurin inhibitors dose reduced	8 (44.4%)	10 (40.0%)	18 (41.9%)	.771
Calcineurin held	1 (5.6%)	0 (0.0%)	1 (2.3%)	.419
Increased baseline steroids	6 (33.3%)	7 (28.0%)	13 (30.2%)	.707
Sirolimus held	2 (11.1%)	0 (0.0%)	2 (4.7%)	.169
COVID‐19 targeted therapy				
Favipiravir	7 (38.9%)	8 (32.0%)	15 (34.9%)	.640
Hydroxychloroquine	8 (44.4%)	2 (8.0%)	10 (23.3%)	.009
Azithromycin	9 (50.0%)	5 (20.0%)	14 (32.6%)	.038
Convalescent plasma	4 (22.2%)	1 (4.0%)	5 (11.6%)	.144
Dexamethasone	11 (64.7%)	7 (35.0%)	18 (48.6%)	.072
Infectious complications				
Any bacterial infection	6 (28.6%)	6 (24.0%)	12 (26.1%)	.725
Bacteremia	1 (5.6%)	0 (0.0%)	1 (2.3%)	.429
Pneumonia	5 (27.8%)	2 (8.3%)	7 (16.7%)	.118
Candidemia	0 (0.0%)	1 (4.0%)	1 (2.2%)	>.99
Outcome during hospitalization				
Required mechanical ventilation	5 (23.8%)	4 (16.0%)	9 (19.6%)	.711
Required HFNC	8 (38.1%)	2 (8.0%)	10 (21.7%)	.028
Required renal replacement therapy	2 (9.5%)	1 (4.0%)	3 (6.5%)	.585
Required inotropes/vasopressors	3 (14.3%)	2 (8.3%)	5 (11.1%)	.652
Required ICU stay	11 (52.4%)	4 (16.0%)	15 (32.6%)	.009
Hospital death	3 (14.3%)	1 (4.0%)	4 (8.7%)	.318
Length of stay*	9.6 ± 7.4	20.7 ± 11.7	12.0 ± 9.2	<.001
	*Mdn* 14 (IQR 7)	*Mdn* 5 (IQR 9)	*Mdn* 10 (IQR 11)	

*Note*: Data were presented as number and percentage unless mentioned otherwise.

Abbreviations: HFNC, high flow nasal cannula; ICU, intensive care unit.

* Mean and standard deviation.

Table 3 shows laboratory markers and oxygen requirements on the days of tocilizumab administration (Days 0 and 3 after receiving the first dose of tocilizumab). Except for CRP, all laboratory measures were generally increasing during the course of the disease. The need for oxygen was generally decreasing during the course of the disease and the requirement for mechanical ventilation.

**Table 3:** Laboratory markers and oxygen requirements at Days 0 and 3 after receiving tocilizumab

	Day 0		Day 3		
	*N*	Value*	*N*	Value*	*p *value**
Laboratory measurements					
WBC (×109/L)	19	5.79 ± 5.15	21	8.73 ± 8.05	.349
Absolute lymphocyte count (×109/L)	13	0.51 ± 0.60	9	0.70 ± 0.59	.575
Lactate dehydrogenase (U/L)	18	322.6 ± 110.8	18	387.6 ± 151.5	.007
CRP (mg/L)	20	140.6 ± 97.3	19	40.1 ± 41.5	<.001
Procalcitonin (ng/ml)	14	2.29 ± 7.49	11	2.55 ± 8.04	.005
ESR (mm/h)	1	39.0 ± 0.	0	‐	‐
Ferritin (μg/L)	21	1619 ± 2806	19	1341 ± 918	.658
d‐dimer (mg/L)	16	1.10 ± 0.74	16	1.15 ± 1.07	.300
Creatinine (umol/L)	21	139.1 ± 114.1	21	150.5 ± 146.7	.737
AST (U/L)	19	26.2 ± 12.1	20	28.6 ± 9.5	.305
ALT (U/L)	19	20.0 ± 14.5	20	25.7 ± 16.0	.009
O_2_ requirement***					
Room air	6	28.6%	7	33.3%	.857
1–5 L/min	9	42.9%	7	33.3%	
6–10 L/min	0	0.0%	0	0.0%	
11–15 L/min	1	4.8%	1	4.8%	
HFNC	3	14.3%	5	23.8%	
Mechanically ventilated	2	9.5%	1	4.8%	

Abbreviations: ALT, alanine aminotransferase; AST, aspartate aminotransferase; CRP, C‐reactive protein; CRS, cytokine release syndrome; ESR, erythrocyte sedimentation rate; HFNC, high flow nasal cannula; N, number.

* Data were presented as mean and standard deviation unless mentioned otherwise.

** Number and percentage.

*** *p* value was derived from Wilcoxon signed ranks test.

## 4 DISCUSSION

Our retrospective study was conducted on patients who were not ventilated and received low flow oxygen aiming to evaluate the early use of tocilizumab in solid organ transplant patients with COVID‐19 pneumonia. The results of our study showed that there was no difference in mortality between patients who received tocilizumab versus those in the standard of care group. Patients in the tocilizumab arm had a shorter hospital stay and required more assisted ventilation than patients in the standard of care group who were not treated with tocilizumab. Besides, patients who received tocilizumab were older, had more severe disease, higher inflammatory markers, and were expected to have worse clinical outcomes than those who received standard of care. In a previous retrospective matched control study, there was no difference in mortality between solid organ transplant patients who received tocilizumab versus standard of care. Most patients in that study required high flow oxygen and were ventilated.^23^

The mortality rate in our study was comparable to another study from Saudi Arabia. In our study, 46 solid organ transplants were evaluated, 45% received tocilizumab, 19% required ventilation, and the overall mortality was 8%. In the other study, 67 solid organ transplants were evaluated, 23% received tocilizumab, 14% required ventilation, and the overall mortality rate was 4%.^24^ The mortality rate of solid organ transplants in Saudi Arabia is lower than in other countries. In a multicenter cohort study in the United States, 480 patients were studied, 13% received anti‐IL‐6 receptor monoclonal antibody, 31% required ventilation, and the overall mortality of solid organ transplants was 20%.^25^

In our study, there was no difference in infectious complications between patients who received tocilizumab versus standard of care, and most infections were bacterial pneumonia. Patients in the current study were followed until discharge or death while hospitalized. The results showed only one episode of candidemia that was documented in the standard of care group. Similar results were described in the retrospective matched control study.^23^ High‐dose steroids were used in 64%, and 72% of patients in our study and the retrospective matched control study, respectively. The concomitant use of steroids and tocilizumab did not increase the risk of infections in solid organ transplant recipients. Most of our patients did not have any rejection episodes within the year before the diagnosis of COVID 19, which would increase their risk of infections following the use of tocilizumab.

In our study, at Day 3 post‐tocilizumab infusion, there was a significant reduction in CRP level, but the level of ferritin and d‐Dimer remained elevated. Various inflammatory parameters were studied in ICU and non‐ICU solid organ transplant recipients. Although patients in the ICU had higher CRP levels, ferritin, and d‐Dimer than non‐ICU patients, CRP was an important inflammatory parameter to monitor disease progression, unlike ferritin and d‐Dimer, which remained elevated.^26^ It is clinically expected that CRP would fall after tocilizumab administration, however, the data analysis in the current study showed no association between the magnitude of the drop in CRP after tocilizumab and patients' length of hospital stay.

The findings of the current study should be interpreted taking into account its limitations. Our study was limited by the small number of patients, nonstandardized antiviral protocol, and retrospective nature of the study. First, retrospective observational studies cannot draw cause/effect inferences taking into full consideration its known and unknown confounders. The tocilizumab in the current study was used in patients with severe COVID‐19 infection and had evidence of a higher level of inflammation compared to the standard treatment group. Therefore, the findings should be considered preliminary taking into account the study limitations and, therefore, tocilizumab efficacy should be validated in adequately powered clinical trials with a randomized allocation of treatment. A recent randomized trial showed increased mortality related to tocilizumab when used for ventilated patients.^27^ Future randomized trials are needed to evaluate the efficacy of tocilizumab in solid organ transplant recipients and to determine the optimal timing of administration for a favorable outcome.

## 5 CONCLUSION

In conclusion, the current study findings present some assessment of tocilizumab efficacy in the management of severe COVID‐19 infection which requires mechanical ventilation support and stay in the ICU. The tocilizumab group was a sicker group of patients, and yet mortality was not increased in this group, and hospital stay was shorter. Furthermore, the overall mortality reported by these two centers was far lower than in most other case series or registries of SOT/COVID, so it is even possible that this use of tocilizumab could have contributed to these favorable outcomes. While this cannot approach the level of evidence of a randomized trial, it does suggest that there could be some benefit of tocilizumab, especially in the group of patients who are rapidly progressing and are in the early upswing of the inflammatory phase, but before full‐blown respiratory failure has occurred. As COVID‐19 infection is spreading dramatically all over the globe leading to increased inflammatory markers, irreversible lung damage, demand for ventilatory support and mechanical ventilation, and dramatic radiological manifestations, tocilizumab can be considered a therapy if other treatments are not available or fail to manage the severely ill patients with COVID‐19.

## CONFLICT OF INTERESTS

The authors declare that there are no conflict of interests.

## AUTHOR CONTRIBUTIONS

*Research design*: Amani H. Yamani, Basem M. Alraddadi. *Performance of research and data acquisition*: Amani H. Yamani, Afnan A. Amer, Fatimah S. Mehdawi, Mohammed A. AL‐Hamzi, Meshari S. Aldajani. *Data analysis and interoperation*: Amani H. Yamani, Basem M. Alraddadi, Aiman M. Ramadan, Abeer N. Al Shukairi. *Writing of paper*: Amani H. Yamani, Majda S. Alattas, Abeer N. AlShukairi, Aiman M. Ramadan, Abbas Al Mutair. *Critical review of paper*: Amani H. Yamani Basem M. Alraddadi, Reem S. Almaghrabi, Ghassan Y. Wali, Abeer N. Al Shukairi, MD, Abbas Al Mutair.
